# Generational differences in the physical activity of UK South Asians: a systematic review

**DOI:** 10.1186/s12966-015-0255-8

**Published:** 2015-07-19

**Authors:** Prachi Bhatnagar, Alison Shaw, Charlie Foster

**Affiliations:** British Heart Foundation Centre on Population Approaches for Non-Communicable Disease Prevention, Nuffield Department of Population Health, University of Oxford, Old Road Campus, OX3 7LF Oxford, UK; Nuffield Department of Population Health, University of Oxford, Old Road Campus, OX3 7LF Oxford, UK

**Keywords:** South Asian, Physical activity, Review

## Abstract

**Background:**

South Asians are some of the least active people in the UK, but we know very little about how physical activity varies within and between different UK South Asian groups. There is much socio-economic and cultural heterogeneity among UK Indians, Pakistanis and Bangladeshis, and the same approaches to increasing physical activity may not be appropriate for all people of these ethnic groups. We report on the variation in physical activity behaviour prevalence in quantitative studies and the variations in attitudes, motivations and barriers to physical activity among South Asians in qualitative papers.

**Methods:**

We performed systematic searches in MEDLINE, Embase and Psychinfo for papers written in English and published between 1990 and 2014. We also attempted to search literature not published in peer-review journals (the ‘grey’ literature). We reported data for the quantitative observational studies and synthesised themes from the qualitative literature according to age-group. We assessed the quality of studies using a National Institute of Health and Clinical Excellence tool.

**Results:**

We included 29 quantitative papers and 17 qualitative papers. Thirteen papers reported on physical activity prevalence in South Asian children, with the majority comparing them to White British children. Four papers reported on adult second-generation South Asians and the rest reported on South Asian adults in general. Second-generation South Asians were more active than the first-generation but were still less active than the White British. There were no high quality qualitative studies on second-generation South Asian adults, but there were some studies on South Asian children. The adult studies indicated that the second-generation might have a more favourable attitude towards physical activity than the first-generation.

**Conclusions:**

There is clear variation in physical activity levels among UK South Asians. Second-generation South Asians appear to be more physically active than the first-generation, but still less active than the White British. More qualitative research is needed to understand why, but there are indications that second-generation South Asians have a more positive attitude towards physical activity than the first-generation. Different strategies to increase physical activity may be needed for different generations of UK South Asians.

## Background

Ethnicity is poorly measured in epidemiological studies, failing to reflect both its social nature and socio-economic and cultural heterogeneity within ethnic groups. Ethnicity is a multifaceted concept encompassing factors such as language, marriage patterns and common ancestry, but this is not accurately reflected by the current measurement methods, such as those used in the national census. Consequently, when we aim to understand physical activity in ethnic groups, we may miss the influence of other factors related to having an ethnic minority background, such as country of birth or the country where most schooling took place. In this paper we review the literature on physical activity prevalence and attitudes within the UK Indian, Pakistani and Bangladeshi populations to explore how physical activity varies between and within these UK South Asian groups.

There have been two reviews on physical activity among UK South Asians during the past decade. Fischbacher et al. 2004 [1] reviewed physical activity in the UK South Asian population, and Babakus & Thompson 2012 [2] reviewed physical activity in South Asian women internationally. Both reviews reported low levels of physical activity among South Asians, with women in particular having a low level of physical activity. Babakus & Thompson 2012 also reported on the barriers and motivations for physical activity in South Asian women found in the literature. While providing valuable information, neither of these reviews discussed how physical activity prevalence varies within different South Asian groups.

Within the UK, the Health Survey for England 2004 was the latest health survey to boost the ethnic minority sample, therefore providing the most reliable estimates for physical activity levels in English ethnic minorities. According to the old physical activity guidelines in place in 2004, 37 % of men and 25 % of women in the general population were doing the recommended levels of physical activity. Comparisons between ethnic minorities show that Bangladeshi men and women had the lowest proportion of people meeting the guidelines (26 % and 11 % respectively) [3]. The Health Survey for England also reported that differences between younger and older people were greater for Indian women and for Bangladeshi respondents than for the general population (mostly the White British group). 18 % of Indian women aged 16 to 34 compared to 2 % of Indian women aged over 55 were highly active [3].

There are no ethnic-specific cohorts in the UK, although there are studies with a high-proportion of ethnic groups in their sample. However, these cohorts are all set up to explore the health and behaviour of children, and information on the health behaviours of UK-born ethnic minority adults will not be available for some years. Since children’s behaviour is heavily influenced by parental behaviour, it is necessary to study adults in order to obtain an accurate picture of how the second-generation differs from their parents.

It is particularly important to understand the differences in risk factors for cardiovascular disease (CVD) between migrant (first-generation) and second-generation ethnic groups because the second-generation of some ethnic groups is still relatively young and may still have a good chance of altering their CVD risk in later life [4]. A childhood in the UK has exposed second-generation ethnic minorities to many experiences that differ from those of their parents, making it plausible that second-generation ethnic minorities also differ from their parents in their health behaviours. Physical activity is known to be low among South Asians compared to the rest of the population [5]. However, exposure to activity in school, through the media and the wider social environment may mean that second-generation ethnic groups will have physical activity patterns that differ from those of the first-generation.

## Methods

We performed systematic searches in MEDLINE, Embase and Psychinfo for papers written in English and published between 1990 and 2014. We also attempted to search literature not published in peer-review journals (the ‘grey’ literature). The website www.better-health.org.uk has a comprehensive list of resources and organisations that work on ethnicity and health; we searched through the website of each organisation that worked with a relevant ethnic group or on physical activity to find additional papers.

We combined three groups of search terms using the ‘AND’ command. The search terms related to South Asian ethnicity were, ‘Asian’, ‘Indian’, ‘Pakistani’ and ‘Bangladeshi’. The terms on physical activity included ‘physical activity’, ‘exercise’, ‘walking’, ‘leisure’, ‘sports’ and ‘transport’. The third group limited the search to the United Kingdom, and the MeSH term ‘United Kingdom’ was used with all its subheadings.

Both MeSH terms and keywords were used; the full search strategy used to search the databases is available on request. We assessed the quality of the papers after determining whether they met the inclusion/exclusion criteria.

The inclusion and exclusion criteria for the reviews were as follows:

Inclusion/exclusion criteria for all:

- Papers must report on South Asian populations residing within the UK (Indian, Pakistani or Bangladeshi).
- Papers must have been published between 1990 and 2014.
- Review articles will be excluded.
- Patient groups will be excluded.

For the quantitative literature:

- Papers must report on the prevalence of physical activity.
- Papers reporting only physiological data on fitness will be excluded.
- Papers reporting observational studies will be included.
- Papers reporting experimental studies will be excluded.

For the qualitative literature:

- Papers must report on the attitudes, influences, barriers or motivators to physical activity.

**Fig. 1:**
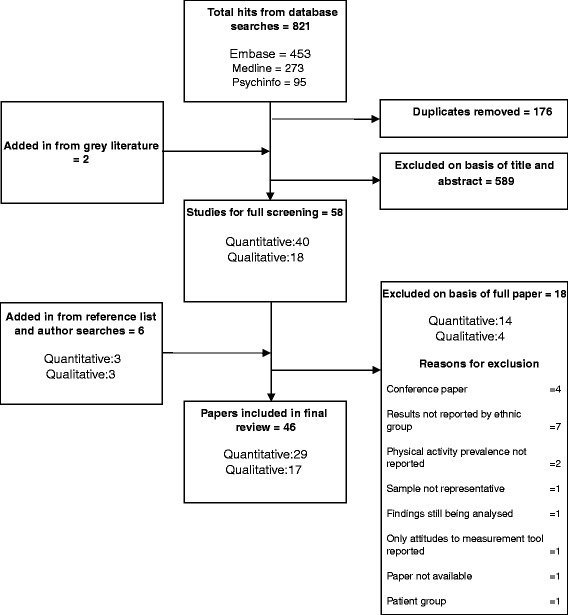
Flow chart of review process

The search yielded 821 hits altogether. We reviewed the search results by first examining the titles and abstracts of papers that met the inclusion criteria. If the abstract met the inclusion criteria, we read the paper in full to decide whether or not to include it in the review. CF read through 20 % of the abstracts to judge against the inclusion/exclusion criteria and any disagreements were resolved through discussion with PB. We searched the references of all papers read in full and the publication lists of included authors; we subjected these to the same title and abstract screening process. Figure 1 illustrates the review process. Reasons for exclusion at the title and abstract level included not reporting on the prevalence of physical activity, not reporting on South Asian ethnic groups and not reporting on a UK population.

### Data extraction

For the quantitative studies, we extracted data on ethnic group, whether there was a comparison to the White British or general population, type of measurement, location of study and sample size. For the qualitative studies we extracted information on data collection method (focus groups or interviews), ethnic group, location, number of participants and analytical approach.

### Study quality assessment

We assessed the quality of the papers included in the review by using a quality appraisal tool from the National Institute for Health and Clinical Excellence (NICE) Public Health guidance [6]. Quality appraisal tools are intended as guides; we used the NICE checklist for studies reporting correlations and associations in order to assess both the internal and external validity of the papers.

#### Quantitative studies

For the quantitative observational studies, we organised the reported prevalence of physical activity according to age-group. For the qualitative literature we synthesised the themes and also reported these according to age-group.

Table 1 describes the basic characteristics of the included papers, of which there were 29 [7–35]. One study was a prospective cohort [13] and the rest were cross-sectional. Eight studies had participants from central England, six were in London, five were based in the North of England, four in Scotland, one in Bristol and the rest were across England or the UK. The age of participants ranged from two to 79 years, and we present the results for children and adults separately. Twelve of the 29 papers reported by sub-group of South Asian ethnicity or religion, with the rest grouping Indians, Pakistanis and Bangladeshis into the broader South Asian category. All but one of the included studies compared the physical activity prevalence of South Asians to the White British, therefore we have included the comparison to the White British group in order to contextualise the reported activity of second-generation South Asian groups.

**Table 1:** Characteristics of quantitative studies

Author and year	Study design	South Asian Ethnic groups (N)	Comparator (N)	Sex	Age-group	Location	Physical activity measurement instrument
[7] Duncan et al. 2006	Cross sectional	Asian (67)	White (176), Black (33)	Male and Female	11 to 14	Birmingham	Self-report, validated questionnaire - Four by one day
[8] Duncan et al. 2008	Cross sectional	South Asian (209)	White (397)	Male and Female	11 to 14	Birmingham	Self-report, validated questionnaire - Four by one day
[9] Duncan et al. 2012	Cross sectional	South Asian (67)	White (469)	Male and Female	8 to 11	Coventry	Pedometers worn over 4 days
[10] Eyre et al. 2013	Cross sectional	South Asian (65)	White European (96)	Male and Female	8 to 9	Coventry	Physical activity and heart rate worn monitor for 7 days
[11] Falconer et al. 2014	Cross sectional	South Asian (607)	White (1,904), Black/Black British (226)	Male and Female	4 to 5 and 10 to 11	5 PCTs in England	Self-report questionnaires
[12] Ghouri et al. 2013	Cross sectional	South Asian (87)	European (99)	Male	40 to 70	Scotland	Accelerometers worn for 7 days
[13] Griffiths et al. 2013	Prospective cohort	Indian (139), Pakistani (177), Bangladeshi (70)	White (5,710), Mixed (168), Black (142), Other (90)	Male and Female	Age 7	UK	Accelerometer worn for 7 days, during waking hours.
[14] Harding et al. 2008	Cross sectional	Indian (218) Pakistani/Bangladeshi (222)	White UK (589), White Other (218), Black Caribbean (453), Black African (593), Mixed (279)	Male and Female	11 to 13	London	Self-report physical activity questions on vigorous sports
[15] Hayes et al. 2002	Cross sectional	Indian (249), Pakistani (287), Bangladeshi (117)	European (749)	Male and Female	25 to 74	Newcastle	Self-report physical activity questionnaire, then created an index
[16] Hemmings et al. 2011	Cross sectional pilot	South Asian (12)	British White (11)	Male	14 to 15	London	Accelerometer worn for 7 days
[34] Hine et al. 1995	Cross sectional	Indian (52), Pakistani (79), Bangladeshi (21)	None	Female	18 to 74	Bristol	Self-report questionnaire
[17] Karlsen et al. 2010	Cross sectional	Muslim Indian (270), Pakistani (2,120), Bangladeshi (1,943), Sikh (657), Hindu (1,195)	Christian by White British (10,577), Irish (1,729), Caribbean and No religion by White British (2,371), Caribbean (309)	Male and Female	Above 2	England	Self-report question on taking no regular physical activity
[18] Khunti et al. 2007	Cross sectional	South Asian (2,732)	White European (447)	Male and Female	11 to 16	Leicester	Self-report questionnaire derived from Four by one day
[19] Knight et al. 1993	Cross sectional	Asian (128)	Non-Asian (160)	Male	20 to 65	Bradford	Self-report lifestyle questionnaire including exercise
[20] Lean et al. 2001	Cross sectional	South Asian (119)	Italian (90), General Population (50)	Female	20 to 42	Glasgow	Self-report question on sport and recreational exercise
[21] McMinn et al. 2011	Cross sectional	South Asian (487)	White European (508), Black African-Caribbean (576)	Male and Female	9 to 10	London, Birmingham, Leicester	Accelerometer worn for 7 days, during waking hours.
[22] De Munter et al. 2012	Cross sectional	Indian (1,264)	English European (14,723), English African (1,112)	Male and Female	35 to 64	England	Self-report questionnaire based on Allied Dunbar National Fitness Survey
[23] Owen et al. 2009	Cross sectional	South Asian (494)	White European (562), Black African-Caribbean (607)	Male and Female	9 to 10	London, Birmingham, Leicester	Accelerometer worn for 7 days, during waking hours.
[24] Pollard et al. 2008	Cross sectional	British Pakistani (60)	European (25)	Female	20 to 40	North East England	Self-report, validated questionnaire -International Physical Activity Questionnaire
[25] Pollard et al. 2012	Cross sectional	British Pakistani (67)	White British (70)	Female	9 to 11	North East England	Accelerometer worn for 2 school days
[26]Pomerleau et al. 1999	Cross sectional	South Asian (291)	European (559), Afro-Caribbean (303)	Female	40 to 69	London	Self-report questionnaire
[27] Riste et al. 2001	Cross sectional	Pakistani (132)	European (471), African-Caribbean (316)	Male and Female	35 to 79	Manchester	Self-report validated questionnaire
[28] Smith et al. 2012	Cross sectional	Indian, Pakistani, Bangladeshi	White	Male and Female	16 to 55	England	Self-report questionnaire based on Allied Dunbar National Fitness Survey
[29] Williams et al. 1994	Cross sectional	South Asian (173)	General population (344)	Male and Female	30 to 40	Glasgow	Self-report questionnaire.
[30] Williams et al. 1998	Cross sectional	British Asian (334)	Other origin (490)	Male and Female	14 to 15 in South Asians Age 35 in General population	Glasgow	Self-report questionnaire.
[35] Williams et al. 2010	Cross sectional	South Asian Sikhs (571), Muslims (179), Hindus (315)	Whites (818)	Male and Female	35 to 75	London	Self-report, validated questionnaire -International Physical Activity Questionnaire
[32] Williams et al. 2011 - JECH	Cross sectional	South Asian (5,421)	Whites (8,974)	Male and Female	Over 16	England	Self-report questionnaire based on Allied Dunbar National Fitness Survey
[31] Williams et al. 2011 - Heart	Cross sectional	Indian (1,244), Pakistani/Bangladeshi (876)	White (13,293)	Male and Female	Over 16	England	Self-report questionnaire based on Allied Dunbar National Fitness Survey
[33] Yates et al. 2010	Cross sectional	South Asian (1,164)	White European (4,310)	Male and Female	25 to 75 in South Asians 40 to 75 in White European	Leicester	Self-report, validated questionnaire -International Physical Activity Questionnaire

Eight of the studies used accelerometers or pedometers to objectively measure physical activity levels, and six of these studies were carried out with school-children. Six studies reported using validated self-report questionnaires. However the remaining majority of papers used self-report, non-validated questionnaires to measure physical activity.

#### Qualitative studies

We included 17 studies in this review [36–52] (Table 2). The locations of the studies varied: four were based in London, four in the North West of England, four in central England and the remaining studies were conducted in Scotland, Wales or Great Britain in general. The age range of participants ranged from eight to 70 years, and we present the findings for children and second-generation adults, using the socioecological model; results for adults of any age are in the appendix. Six of the studies reported interviewing South Asians, with the other 11 presenting findings for Indians, Pakistanis or Bangladeshis separately. Interestingly, none of the papers included here specifically reported differences between South Asian groups, usually just stating when a finding or theme was relevant to one of the ethnic groups in particular.

**Table 2:** Characteristics of qualitative studies

	South Asian Ethnic group	Location of study	Main focus of study	Data collection method	Number of participants	Sex of participants	Age groups	Reporting of adult second-generation information?
[36] Brophy et al. 2011	Bangladeshi	South Wales	Asking teenager to suggest recommendation to increase their physical activity	4 Focus groups	24	Male and Female	16 to 18	No
[37] Eastwood et al. 2013	South Asian	London	Assessing the risk of CVD in religious and community settings	Semi-structured interviews	24	Male and Female	30 to 67	No
[38] Farooqi et al. 2000	South Asian	Leicester	Understanding attitudes and knowledge of lifestyle risk factors for CHD	6 Focus groups	44	Male and Female	Over 40	Yes
[39] Grace et al. 2008	Bangladeshi	London	Preventing diabetes	17 Focus groups and Semi-structured interviews	129 in Focus groups 8 interviews	Male and Female	Mean age 35	Yes
[40] Horne et al. 2009	South Asian	North West England	Fall prevention in 60 to 70 year olds	Participant observation, 15 Focus groups and Semi-structured interviews	87 in Focus groups 40 interviews	Male and Female	60 to 70	No
[41] Horne et al. 2010	South Asian	North West England	Influence of primary health-care professionals on physical activity	Participant observation, 15 Focus groups and Semi-structured interviews	87 in Focus groups 40 interviews	Male and Female	60 to 70	No
[42] Horne et al. 2012	Indian and Pakistani	North West England	To identify the attitudes and beliefs associated with the uptake and adherence of physical activity among community-dwelling South Asians	Focus groups and Semi-structured interviews	29 in Focus groups 17 interviews	Male and Female	In 60s	No
[43] Horne et al. 2013	Indian and Pakistani	North West England	Understanding the barriers to maintaining physical activity	15 Focus groups and Semi-structured interviews	29 in Focus groups 17 interviews	Male and Female	In 60s	No
[44] Jepson et al. 2008 (report)	Indian, Pakistani, Bangladeshi	Aberdeen, Glasgow, Edinburgh	To explore the barriers, facilitators and motivators for South Asians	9 Focus groups and Semi-structured interviews	59 in Focus groups 10 interviews	Male and Female	Ages 20 to 40	No
[45] Jepson et al. 2012	Indian, Pakistani, Bangladeshi	Aberdeen, Glasgow, Edinburgh	Describing the types of and motivators for physical activities that South Asians do	9 Focus groups and Semi-structured interviews	59 in Focus groups 10 interviews	Male and Female	Unknown	No
[46] Johnson 2000	Indian, Pakistani, Bangladeshi	Midlands	To fill the gap in knowledge about ethnic minority lifestyles and health	Not reported	Not reported	Male	Unknown	No
[47] Khunti et al. 2008	South Asian	Leicester	Understanding the impact of an Action Research Partnership to prevent diabetes	18 Focus groups	Not reported	Male and Female	11 to 15	No
[48] Pallan et al. 2012	South Asian	Birmingham	Understanding the contextual influences on obesity	9 Focus groups	68	Male and Female	Unknown (adults talking about children)	No
[49] Rai & Finch 1997	Indian, Pakistani, Bangladeshi	England	To investigate attitudes towards and barriers to physical activity in South Asian and Black communities in England	14 Focus groups	109	Male and Female	18 to 50	Yes
[50] Rawlins et al. 2013	Indian, Pakistani, Bangladeshi	London	Perceptions of healthy eating and physical activity in young children	13 Focus groups	70	Male and Female	8 to 13	No
[51] Rogers et al. 1997	Bangladeshi	London	To describe the contributing factors to variations in health-related behaviours and attitudes in 12 year olds	Semi-structured interviews	41	Male and Female	Age 12	No
[52] Victor 2014	Bangladeshi and Pakistani	Great Britain	Physical activity during the daily life of elders	Semi-structured interviews	109	Male and Female	Over 50	No

## Quality assessment

### Quantitative papers

Of the papers reporting on children, only one reported a power calculation for the sample size [30]. The other 11 papers provided no justification for their sample size; the majority only reported the number of people they had recruited, implying that their aim was to recruit as many children as possible into their studies. Three of the studies on children had fewer than 70 South Asian participants [7, 9, 10]. Without a sample size calculation, we cannot know whether there are enough participants to detect a particular size of difference between the two ethnic groups and therefore whether to reject the null hypothesis of no difference between the two ethnic groups. The fact that all of the studies do report a difference, despite using different measurements, indicates that rejection of the null hypothesis may be the correction conclusion, but without a sample size calculation we cannot be sure.

There were also issues with the quality of measurement, both for ethnicity and physical activity. For the studies on children, ethnicity was measured through self-identification to pre-defined categories [9, 13, 14, 18, 21, 23, 25] (often through the parents) or through school records, where ethnicity is reported by the parents [7, 8, 10, 11, 30]. While self-identifying ethnicity is regarded as the best measure, assigning people to pre-defined categories or asking them to choose from a pre-defined list restricts the process of self-identification, and therefore the meaning of ethnicity.

Five of the 12 studies on children measured physical activity using an objective measurement tool, either an accelerometer or a pedometer [9, 10, 13, 21, 23], and the rest used a self-report physical activity questionnaire or, in one case, a single physical activity question [30]. While objective measures of physical activity are more reliable than questionnaires, which are subject to recall bias or social desirability bias, particularly for children [53], there are still potential pitfalls. The placement of the accelerometer on the body (at the hip, ankle or wrist) may affect the output of the device, and the devices cannot be worn during wet activities such as swimming. Although accelerometers and pedometers are able to provide objective data on physical activity, a criticism of them is that they are unable to measure the context in which the physical activity is carried out. Of the studies using questionnaires, three used a validated questionnaire [7, 8, 18] and one used a non-validated questionnaire on vigorous sports [14].

Of the 12 studies on children, only four included socioeconomic or deprivation factors in their analyses [9, 11, 13, 14]. This is surprising given that socioeconomic factors and neighbourhood deprivation are common explanations for why South Asians do less physical activity than the White British population [54]. The exclusion of these factors from analyses that account for other potential confounding factors, such as age and sex, points to the lack of theoretical models in the papers. No paper, on either children or adults, reported using a theoretical model to describe the potential causes of physical inactivity in populations. By not referring to any theoretical model, the authors have excluded a potential confounder from their analyses – which they had often measured as part of the study. The exclusion of socioeconomic status or deprivation means that it is possible that the differences found between ethnic groups could be due to their socioeconomic circumstances rather than to their ethnic background.

#### Second-generation adults

Of the four papers reporting on second-generation South Asian adults, three lacked power calculations [24, 28, 32], rendering it impossible in these cases to determine whether the sample sizes obtained were large enough to detect a difference in prevalence between generations, or between the UK-born ethnic groups and the White British ethnic groups. One of the four papers reported a sample size calculation [20]. The other three papers reported either using existing survey data or simply recruiting as many participants as possible. Low sample sizes may mean that the samples are biased towards a particular age group, sex or other characteristic and may not give accurate snapshots of the prevalence of risk factors in second-generation ethnic minorities in the UK. Two of the papers used the snowball sampling technique to increase their sample size [20, 24]. Snowball sampling is commonly used in populations where it is difficult to recruit participants, but produces a biased sample because recruitment is done through the contacts of existing participants. This is likely to affect the generalisabilty of the findings to the wider ethnic minority populations that were studied, although the authors did not discuss this issue.

Three papers used non-validated self-report measures to estimate prevalence [20, 28, 32]. Self-report data is open to recall bias and there is a real possibility of reporting an inaccurate prevalence. No study reporting on second-generation adults used an objective measure of physical activity.

Three papers measured ethnicity through people self-identifying to pre-defined categories. In the fourth paper, the authors obtained ethnicity information by identifying South Asian surnames in birth records [20]. The authors do not report confirming this ethnicity information with their participants, and as a result each participant has had their ethnicity assigned for them, based on ancestry. This is essentially only a physical measure of ethnicity; if people have not self-identified as South Asian, we cannot be sure that they have a cultural or social identification with the South Asian population.

The analysis of two papers was insufficient [20, 24]. Where regression models were conducted, important confounders, such as socioeconomic measures, were not included, even where data were available in the study. As discussed with the studies on children, this highlights the absence of theoretical frameworks addressing all potential causes of the behaviour being studied in the construction of statistical models to explain the behaviour. Not including known confounders affects the conclusions drawn from the study, as any associations found between variables may in fact be confounded by a third variable. The dearth of socioeconomic status measures in the regression models raises concerns about the methods and theories used to identify potential confounders in the studies as a whole.

### Qualitative papers

#### Children

Five papers reported on motivations or barriers to physical activity in South Asian children in the UK [36, 47, 48, 50, 51]. All of these papers were of reasonable quality, but none of the papers discussed how the researchers themselves might have influenced the data collection or analysis, through their own background or attitudes. Reflecting on how an interviewer might influence the interview or focus group is important because interviewees may offer more or less information according to how they perceive the interviewer [55].

Only one study on children reported using an existing theoretical model, which the authors used to guide their analysis of the data [39]. Theoretical models in qualitative research can be used to develop hypotheses, to develop interview guides or, as in this paper, used to guide the analysis [56]. Most papers conducted a thematic analysis of their data, but placing these themes into a theoretical framework is useful for interpretation and can aid understanding of the practical value of the findings.

#### Second-generation adults

Three studies reported information on the attitudes, motivations and barriers to physical activity in second-generation South Asian adults [38, 39, 49]. One of these was a report, rather than a peer-reviewed article and had limitations in the reporting of the methods, context and analysis [49]. The authors did not describe their sampling approach, the context in which focus groups were held or give detail of how they analysed the focus group data. Without this information it is difficult to assess the quality of the study accurately.

None of these studies reported how the researcher may have influenced data collection. Of the three studies, only Grace et al. 2008 reported receiving ethical approval for their study [39]. Ethical approval is important in any research project in order to protect research participants and ensure researchers and universities are carrying out appropriate research. All three studies neglected to discuss the limitations of their studies, a key element of high quality research. Reporting the limitations of your study allows the reader to ascertain whether the authors themselves understands the extent to which their findings are valid, and also allows the reader to gauge the transferability of the findings.

The two peer-review articles provided only fleeting remarks on adult second-generation physical activity attitudes. The report by Rai & Finch 1997 provided some more information, although the information was not reported separately for the second-generation [49]. Poor discussion of the study limitations, analytical approach and methods means that although the findings reported are rich in their description, the only study with detailed information on second-generation South Asian adults in the UK is low in quality.

For the rest of the papers, the overall quality was affected by the fact that all but one [45] failed to discuss the impact of the researcher on the data collection and analysis. Two papers were of fairly low quality [46, 51], with poor reporting of data collection methods, the context in which the research was carried out, the analytical techniques, and no discussion of the study limitations or reporting of whether ethical approval was received. Only one paper reported using a theoretical model [52].

## Results

### Prevalence of physical activity

#### South Asian children

One study on children was a prospective cohort, which collected physical activity data from the children in year seven of their study. The rest of the studies on children were cross-sectional. Seven studies were located in the Midlands, with two of those also recruiting children from London. One study was exclusively based in London, one was based in the North East of England and another in Glasgow. Two studies were conducted throughout the UK or England.

Information on the number of UK-born South Asian children is only available for three studies. Griffiths et al. 2013 used the Millennium Cohort Study, which recruited children born in the UK in the year 2000, therefore all the children included in their paper are second-generation (139 Indian, 177 Pakistani and 70 Bangladeshi) [13]. Harding et al. 2008 report on results from the DASH Study, which is based in London; they report that 76.1 % of the Indian group and 82.9 % of the Pakistani/Bangladeshi group were born in the UK (166 and 184 respectively) [14]. Williams et al. 1998 report that 287 (86 %) of their South Asian sample were born in the UK [30].

Studies reporting on physical activity prevalence among South Asian children all reported a lower prevalence compared to White British children, irrespective of the measurement tool (Table 3). Studies that measured boys and girls separately consistently reported that girls were less physically active than boys, for both South Asian and White British groups.

**Table 3:** Physical activity prevalence in South Asian children

Author and year	Age-group	Location	Physical activity measurement instrument	Main findings – all children	Main findings – Male	Main findings – Female
[13] Griffiths et al. 2013	Age 7	UK	Accelerometer worn for 7 days, during waking hours.	Meeting recommended activity levels:		
				White: 51.4 %		
				Indian: 40.0 %		
				Pakistani: 45.2 %		
				Bangladeshi: 32.8 %		
				Overall counts/minute:		
				White: 597		
				Indian: 511		
				Pakistani: 563		
				Bangladeshi: 538		
				Sedentary hours/day:		
				White: 6.5		
				Indian: 6.9		
				Pakistani: 6.4		
				Bangladeshi: 6.5		
				Moderate and vigorous minutes/day:		
				White: 60.2		
				Indian: 52.6		
				Pakistani: 58.2		
				Bangladeshi: 52.9		
				Steps/day:		
				White: 10,343		
				Indian: 8,699		
				Pakistani: 9,419		
				Bangladeshi: 8,894		
[11] Falconer et al. 2014	4 to 5 and 10 to 11	5 PCTs in England	Self-report questionnaires	Child does not achieve ≥1 hr of physical activity/day		
				White: 56.2 %		
				Asian: 59.4 %		
[10] Eyre et al. 2013	8 to 9	Coventry	Physical activity and heart rate worn monitor for 7 days	Meeting WHO recommended activity levels:		
				White European: 73 %		
				South Asian: 35 %		
				Wake hour average counts/minute:		
				White European: 116		
				South Asian: 102		
				Sedentary hours/day:		
				White European: 15.2		
				South Asian: 15.7		
				Moderate and vigorous hours/day:		
				White European: 1.3		
				South Asian: 1.0		
				Counts/minute during break-time:		
				White European: 341		
				South Asian: 317		
[21] McMinn et al. 2011	9 to 10	London, Birmingham, Leicester	Accelerometer worn for 7 days, during waking hours.	Average counts/minute:		
				White European: 481		
				South Asian: 452		
[23] Owen et al. 2009	9 to 10	London, Birmingham, Leicester	Accelerometer worn for 7 days, during waking hours.	Average counts/minute:	Average counts/minute:	Average counts/minute:
				White European: 498	White European: 537	White European: 463
				South Asian: 457	South Asian: 510	South Asian: 414
				Sedentary minutes/day:	Sedentary minutes/day:	Sedentary minutes/day:
				White European: 554	White European: 551	White European: 556
				South Asian: 593	South Asian: 579	South Asian: 604
				Moderate and vigorous minutes/day:	Moderate and vigorous minutes/day:	Moderate and vigorous minutes/day:
				White European: 70	White European: 77	White European: 62
				South Asian: 65	South Asian: 73	South Asian: 57
				Mean number of steps:	Mean number of steps:	Mean number of steps:
				White European: 10,220	White European: 10,882	White European: 9,660
				South Asian: 9,314	South Asian: 10,202	South Asian: 8,571
[25] Pollard et al. 2012	9 to 11	North East England	Accelerometer worn for 2 school days			Percentage of time sedentary during recess:
						White British: 54.5 %
						British Pakistani: 57.3 %
						Percentage of time in moderate and vigorous activity during recess:
						White British: 15.4 %
						British Pakistani: 12.9 %
[9] Duncan et al. 2012	8 to 11	Coventry	Pedometers worn over 4 days	Average weekday steps/day:		
				White: 14,734		
				South Asian: 13,023		
				Average weekend steps/day:		
				White: 11,135		
				South Asian: 10,383		
				Average total steps/day (PB calculation):		
				White: 12,935		
				South Asian: 11,703		
[14] Harding et al. 2008	11 to 13	London	Self-report physical activity questions on vigorous sports		Percentage in most active 1^st^ quartile:	Percentage in most active 1^st^ quartile:
					White UK: 23.9 %	White UK: 12.6 %
					Indian: 23.8 %	Indian: 16.5 %
					Pakistani/Bangladeshi: 31.3 %	Pakistani/Bangladeshi: 14.9 %
					Percentage in least active 4^th^ quartile: White UK: 21.4 %	Percentage in least active 4^th^ quartile: White UK:38.0 %
					Indian: 18.0 %	Indian: 46.3 %
					Pakistani/Bangladeshi: 17.2 %	Pakistani/Bangladeshi: 38.3 %
[7] Duncan et al. 2006	11 to 14	Birmingham	Self-report, validated questionnaire - Four by one day	Very Inactive:		
				White: 20.9 %		
				Asian: 14.9 %		
				Inactive:		
				White: 43.5 %		
				Asian: 44.8 %		
				Moderately Active:		
				White: 21.5 %		
				Asian: 26.9 %		
				Active:		
				White: 14.1 %		
				Asian: 13.4 %		
[8] Duncan et al. 2008	11 to 14	Birmingham	Self-report, validated questionnaire - Four by one day	Average daily minutes spent in moderate and vigorous physical activity:		
				White: 90.0		
				South Asian: 68.2		
[18] Khunti et al. 2007	11 to 16	Leicester	Self-report questionnaire derived from Four by one day	Light aerobic exercise on six or more days during previous two weeks:	Light aerobic exercise on six or more days during previous two weeks:	Light aerobic exercise on six or more days during previous two weeks:
				White European: 39 %	White European: 42 %	White European: 37 %
				South Asians:40 %	South Asians: 40 %	South Asians: 39 %
				Hard aerobic exercise on six or more days during previous two weeks:	Hard aerobic exercise on six or more days during previous two weeks:	Hard aerobic exercise on six or more days during previous two weeks:
				White European: 41 %	White European: 52 %	White European: 32 %
				South Asians: 37 %	South Asians: 48 %	South Asians: 25 %
[30] Williams et al. 1998	14 to 15	Glasgow	Self-report questionnaire		Physical exercise for 20 minutes once a week or less:	Physical exercise for 20 minutes once a week or less:
					Other origin: 8 %	Other origin: 15 %
					British Asian: 18 %	British Asian: 16 %
					Physical exercise for 20 minutes 2–3 times/ week:	Physical exercise for 20 minutes 2–3 times/ week:
					Other origin: 22 %	Other origin: 39 %
					British Asian: 29 %	British Asian: 53 %
					Physical exercise for 20 minutes 4–6 times/week:	Physical exercise for 20 minutes 4–6 times/week:
					Other origin: 40 %	Other origin: 26 %
					British Asian: 26 %	British Asian: 15 %
[16] Hemmings et al. 2011	14 to 15	London	Accelerometer worn for 7 days		Low activity in counts/minute:	
					British White: 267.4	
					British South Asian: 260.2	
					Moderate activity in counts/minute:	
					British White: 70.5	
					British South Asian: 78.1	
					Vigorous activity in counts/minute:	
					British White: 5.2	
					British South Asian: 5.1	

#### Differences between males and females

Owen et al. (2009) used an accelerometer to measure physical activity in South Asian children and White European children, and also examined differences between boys and girls. South Asians were less physically active than White European children for all measures, but there were large differences between girls and boys for both ethnic groups. South Asian girls were the least active, spending the largest number of minutes being sedentary and the smallest number of minutes being moderately or vigorously active. Harding et al. 2008 combined the Pakistani and Bangladeshi group and found that Pakistani/Bangladeshi girls were the least active compared to the White British and Indian groups. Interestingly, almost a third of Pakistani/Bangladeshi boys were in the most active quartile, compared to around a quarter of Indian and White British boys [14].

Only one study examined Indian, Pakistani and Bangladeshi groups separately [13]. Measured using accelerometers, in this study the Bangladeshi group was the least active and the Indian group the most active (33 % versus 40 % respectively meeting recommended physical activity levels).

#### Activity during break-time

Eyre et al. 2013 used a pedometer and heart-rate monitor to measure activity levels in children aged between eight and nine years. The authors found that South Asian children were less physically active than White European children for all measures included in their study. They found significant differences between the two ethnic groups for the average counts per minute overall and during break-time, with South Asians being less active during break-time [10]. Pollard et al. 2012 examined differences in activity during break time in school, and found that British Pakistani girls were less active than White British children, which concurs with the findings from Eyre et al. 2013 [25].

#### Weekend and Weekday activity

While not reported in Table 3, Eyre et al. 2013 also found that South Asian children were less active than White European children at the weekends and after school [10]. Duncan et al. 2012 report on the average steps per day taken by South Asian children, at the weekend or a weekday. They found that South Asian children were less active than White children both on weekdays and at weekends, which is in line with the results reported by Eyre et al. 2013 [9].

### Second-generation South Asian adults

Four studies reported on the physical activity of second-generation adult South Asian groups [20, 24, 28, 32]. Table 4 shows that while they used different measures, all four reported that second-generation South Asians were more active than the first-generation. The three studies comparing South Asian and White British groups showed that the second-generation is still less active than the White British population.

**Table 4:** Physical activity prevalence in second-generation South Asian adults

Author and year	Sex	Age-group	Location	Physical activity measurement instrument	Main findings
[20] Lean et al. 2001	Female	20 to 42	Glasgow	Self-report question on sport and recreational exercise	Engaging in no sport or recreational exercise:
					Migrant South Asians: 82 %
					British-born South Asians: 77 %
					Italian/General Population: 50 %
[24] Pollard et al. 2008	Female	20 to 40	North East England	Self-report, validated questionnaire -International Physical Activity Questionnaire	Median MET-minutes:
					Migrant British Pakistani: 1,040
					British-Born British Pakistani: 1,626
					European: 2,394
					Median pedometer counts:
					Migrant British Pakistani: 3,371
					British-Born British Pakistani: 3,506
					European: 3,781
[32] Williams et al. 2011 - JECH	Male and Female	Over 16	England	Self-report questionnaire based on Allied Dunbar National Fitness Survey	Mean total METs:
					Male UK-born South Asian: 1,385.23
					Male born outside UK South Asian: 935.53
					Female UK-born South Asian: 972.50
					Female born outside UK South Asian: 843.66
[28] Smith et al. 2012	Male and Female	16 to 55	England	Self-report questionnaire based on Allied Dunbar National Fitness Survey	Three or fewer occasions of moderate/vigorous activity in the past four weeks:
					White: 26.7 %
					First-generation Indian: 43 %
					Second-generation Indian: 31.5 %
					First-generation Pakistani: 50.2 %
					Second-generation Pakistani: 38.5 %
					First-generation Bangladeshi: 60.8 %
					Second-generation Bangladeshi: 49.4 %

Lean et al. 2001 [20] and Smith et al. 2012 [28] both used a single question on physical inactivity to compare migrant South Asians, second-generation South Asians and White British or European origin populations. Both reported that adult second-generation South Asians were more active than the migrant generation, but still less active than the White British or European origin population. Smith et al. 2012 reported on physical activity in Indians, Pakistanis and Bangladeshi separately. The pattern of lower physical inactivity in the second-generation compared to the first-generation was the same for all three ethnic groups, although the actual levels of inactivity were differed between the three groups. Within the South Asian ethnic group, the Indian ethnic group had the lowest prevalence of physical inactivity (31.5 %) and the Bangladeshi group had the highest prevalence of physical inactivity (49.4 %); the White ethnic group had the lowest prevalence of physical inactivity overall (26.7 %). The difference in mean age between the first and second-generation was less than 10 years in both papers.

Pollard et al. 2008 used the International Physical Activity Questionnaire to measure physical activity in Pakistani women, which asks a range of questions [24]. As with the results from Lean et al. 2001 and Smith et al. 2012, the second-generation Pakistani women were more active than the migrant generation, but less active than the European origin population. Pollard et al. 2008 found a significant difference between the groups for median MET-minutes (p = 0.03) but not for median pedometer-counts (p = 0.12). The difference in mean age between the migrant and British-born generation was about 2 years. Williams et al. 2011 also calculated METs from a questionnaire and found significantly higher mean METs in the UK-born South Asians as compared to the migrant South Asians [32].

### Attitudes, motivators and barriers to physical activity

The review of quantitative observational papers revealed that there is not only evidence of variation in physical activity prevalence within UK South Asians, but also between the first and second-generations. The quantitative papers had limited information on potential explanations for those differences. With our review of the qualitative literature we therefore aimed to ascertain what studies had been done exploring the attitudes, beliefs, motivators and barriers towards physical activity in UK South Asians, with a particular focus on the second-generation.

### Attitudes, motivators and barriers for physical activity in children

Five papers reported on motivations and barriers to physical activity in South Asian children, but only two of these discussed generational differences in health behaviours within South Asian groups. Table 5 summarises these themes using the socioecological framework as a guide. Placing the themes into a socioeconomic framework helps to understand if the motivations and barriers are at an individual-level, social-level or neighbourhood-level; once we know this, we can begin to understand in which areas interventions might be necessary. The majority of the themes focused on barriers, which were situated at all levels of the socioeconomic model, including Asian cultural factors, school facilities, the neighbourhood environment and parental concerns over the cost of physical activity.

**Table 5:** Motivators and barriers to South Asian children in the UK

	Religious factors	Asian cultural factors	Western gender factors	Other individual factors	School Facilities	Neighbourhood environment	Economic
Motivators		Family activities	Boys are motivated by their peers and siblings (Bangladeshi boys)				
			Boys are more interested in sports than girls are				
Barriers	Attending the Mosque after school limits time	Pakistani and Bangladeshi parents themselves are inactive	It’s embarrassing to exercise (Bangladeshi girls)	Parents have limited awareness of physical activity recommendations	Lack of changing rooms and storage facilities	Fear of unsafe roads in high socioeconomic groups	Concern about the cost of physical activities
			Lack of interest in PE classes in girls, for all ethnic groups			Concern over security of their children playing outside	
		Being physically active might have a negative effect on their schoolwork (Bangladeshi girls)		Parents who work do not have time to take their children to leisure activities			
						It is quicker and easier to use the car for parents.	
						Lack of facilities in the local area	
		Girls not encouraged to play out because of people looking at them (Bangladeshi)					

Only three themes relating to motivations are present in the literature for children, with two of these focusing on boys. One of the themes was a motivator for boys but a barrier for girls, in that boys were described in one paper as being more interested sports than girls are. One paper also reported that family activities would be more appealing to Asian people and so would help South Asian children be more physically active.

### Attitudes, motivators and barriers around physical activity in second-generation adults

Twelve papers reported on motivators and barriers to physical activity in South Asian adults in the UK, although only three of these reported any information on the second-generation. Two papers and one report described a change in behaviour or attitudes in the second-generation, but gave further no detail. Grace et al. 2008 report that in focus groups, younger and second-generation Bangladeshi women supported resisting the traditional norms and expectations of women in Bangladeshi culture [39]. Farooqi et al. 2000 report that some participants in focus groups commented that, in contrast to the first-generation women being quoted, attitudes among younger Bangladeshi women are changing; for example a participant’s daughter-in-law takes her own children swimming [38]. While only brief, both these papers indicate that there may be a positive change in attitude towards physical activity in the second-generation of South Asian women.

The report by Rai and Finch 1997 describes differences in attitudes between younger and older people; all of their study participants aged under 30 were born in the UK [49]. Rai and Finch 1997 studied both Black and South Asian people and combine the findings from these two ethnic groups in their discussion. They note that younger people do not share some of the beliefs of older people and that their views are more shaped by the media as compared with older people. The authors state that the experience of early life in the UK underpins the differences between the younger and older people, who had grown up in other countries.

While Rai and Finch 1997 have not explicitly reported the differences between second-generation and first-generation South Asians within their report, we were able to analyse the quotes by South Asians aged under 30 (reported as all being born in the UK). Table 6 summarises the themes discussed by participants in both the report by Rai and Finch, and in the two peer-reviewed papers. While there were some Asian cultural and religious barriers, there is an indication that there may be a change in attitudes among the younger and second-generation towards the commonly reported barriers to physical activity found in South Asian women [2]

**Table 6:** Motivators and barriers to adult second-generation South Asians in the UK

	Religious factors	Asian cultural factors	Other individual factors	Local facilities	Economic factors
Motivators		Younger and second-generation women resist traditional norms and expectations of women in Bangladeshi culture	South Asian men described having positive role model as children		
			People like challenges		
			Women want to look good		
			Men want to socialise through activity		
Barriers	Religious activities such as Namaz restricts time	Asian women are more reluctant to use child-minders and so caring for children means they have less time.	Tiredness after work	Lack of facilities in local area	Cost of using facilities
			Young people don’t think about being physically active for health		
	Islam restricts clothing women can wear				
		Experience of racism at gyms			
	Muslim women do not want to use mixed-sex facilities				

## Discussion

### Summary of findings

This review demonstrated that there is some evidence of differences in the prevalence of physical activity between first and second-generation South Asians in the UK, and between second-generation South Asians and the White British population. There is also limited evidence that second-generation South Asian adults have different attitudes to physical activity as compared to the first-generation. The studies that have explored physical activity in children have predominantly focused on barriers, but do also show that factors in the neighbourhood and school environment affect the physical activity of South Asian children in the UK. This is an important finding, as it shows that factors other than ethnic background are affecting the physical activity of South Asian children.

### Comparison to existing literature

Previous reviews on physical activity in South Asians have shown that physical activity prevalence is low in these ethnic groups, but have not reported on variation in prevalence levels within these ethnic groups. While there have been no other reviews on this topic within the UK, there is evidence from the Netherlands to support generational differences in CVD risk factors in ethnic minority groups [57, 58]. Hosper et al. find differences in levels of obesity, smoking, physical activity and alcohol consumption in Turkish and Moroccan migrants to the Netherlands. They also find that socioeconomic status in women was higher in the second-generation compared to the first-generation. While caution must be made in comparing the socioeconomic status changes of two different ethnic groups in two different countries, it is plausible that the advantages of being born in a country compared to migrating to it would benefit the second-generation of an ethnic minority, as appears to be the case for Turkish and Moroccan immigrant women to the Netherlands. This finding corroborates the findings of Smith et al. 2012, who use Health Survey for England data in their paper and also report higher socioeconomic positions in the UK-born ethnic minority groups compared to the first-generation [28]. Unfortunately there was no information available by gender from the papers included in this review to compare to Hosper et al’s findings.

Small samples and failure to adjust for important confounding variables limited the generalisability of results that would have otherwise been highly valuable in ethnicity and physical activity research. Papers studying ethnic minorities can suffer from low sample sizes due to the smaller proportion of people from ethnic minorities in the general population. It is therefore, perhaps, unsurprising if studies reporting on subsections of the UK ethnic minority population struggle to recruit enough participants for a quantitative examination of the pooled results.

The age structure of the second-generation may have made it difficult to obtain a large sample size for second-generation adults. However, some of these studies were conducted over ten years ago and there may now be enough adults in the UK-born ethnic minority groups to improve sample sizes. It should also be noted that the age structure of ethnic minority populations varies for each group, with the average age at migration to the UK and time since the majority of migration took place for each ethnic group affecting the current age structure of the second-generation.

Results from this review indicate that there is some evidence of UK-born ethnic minorities obtaining a higher socioeconomic position than their parents. This is coupled with an increase in physical activity prevalence between the generations. Smith et al’s 2012 paper indicates that higher socioeconomic status may be the cause of changes in obesity prevalence for some ethnic minorities [28], but much more information is needed before accurate recommendations for policy can be made. What we do know is that the epidemiology of CVD in twenty to thirty years’ time is likely to be different for second-generation ethnic minorities if their physical activity behaviours are not the same as those of the migrant generation.

At present a limited amount is known about the epidemiology of physical activity in second-generation South Asians, although it is apparent that they are more active than the migrant generation; even less is known about the reasons for this increase and in what ways physical activity behaviour has changed. More theoretically-informed quantitative research with adequate sample sizes needs to be carried out in order to establish firmly the differences in physical activity prevalence between the generations of ethnic minorities. Future research also needs to distinguish different South Asian minorities to reflect the heterogeneity of the groups comprising ‘South Asian’; appropriate strategies for physical activity improvement can only be developed if research results are presented separately for each ethnic group.

Grieser et al. 2006 explored the physical activity attitudes, preferences and practices of African American, Hispanic and Caucasian girls aged 11 to 13 in the United States [59]. While the authors do not state whether the participants were born in the United States, we do know that the girls were attending school in the United States. The authors found few differences in attitudes towards physical activity between the ethnic groups, and thought that only a small number of these differences were related to ethnic background; for example, many Hispanic girls reported doing childcare in the past seven days. There were differences in the favoured activities of the ethnic groups. Although Grieser et al. 2006 studied different ethnic groups in a different country, the principle that ethnic minority adolescents may have similar physical activity attitudes to the majority ethnic group, or ethnic groups is important to note. The report by Rai & Finch highlights the importance of a childhood in the UK, citing the importance of childhood experiences in developing attitudes towards physical activity [49]. Some of the motivators and barriers to physical activity described by Rai & Finch are similar to those described in a review by Allender et al. 2006. Their review on understanding participation in sport and physical activity in the UK reports issues such as cost and being motivated by wanting to maintain appearance [56]. There is not enough information from this review to know whether second-generation South Asians have similar physical activity attitudes to the White British population in the UK, but if childhood and school experiences are relevant for physical activity attitudes, this is theoretically possible.

### Strengths and limitations of the review

As far as we are aware, this is the first paper to review and assess studies reporting on physical activity in second-generation UK South Asians. We conducted a systematic searched of the literature although we may have missed research published in the ‘grey’ literature.

Ideally, systematic reviews are conducted independently by two researchers whose their findings are compared, but one author carried out the systematic search for this review. We are confident that all relevant papers were included, but because only one person screened the papers for inclusion we cannot rule out the possibility that extra papers may have been eligible for inclusion in the study.

Our analysis of the papers was hindered by the different groupings of ethnic minorities used in the paper. A number of the papers used the broad categorisation of ‘South Asian’, which is not directly comparable to the more detailed categories of ‘Indian’, ‘Pakistani’ and ‘Bangladeshi’ as these three groups differ in their socioeconomic and CVD risk factor profiles [54]. While we attempted to compare all the papers, this should be taken into account.

One strength of this paper is the inclusion of both quantitative and qualitative literature. Quantitative methods are limited in their ability to explain the causes of differences between populations. Qualitative methods are ideally suited to this, and the papers in this review indicate that second-generation South Asians have a different attitude towards physical activity as compared to the first-generation, something which could not be gleaned from the quantitative literature.

## Conclusions

This research highlights that there is variability in physical activity behaviour within South Asians in the UK. This review has shown that for second-generation South Asians, particularly adults, research that accurately measures the level of physical activity is still to be done. Some work has begun on identifying factors that influence physical activity, but in the majority of the quantitative literature, socioeconomic factors are omitted from analysis, and we found no peer-reviewed qualitative literature studying physical activity in second-generation South Asian adults as a distinct group. There were some studies on South Asian children, but these were limited in that they mainly focused on barriers to physical activity.

From the papers found in this review, it seems there is a significant gap in the literature on the perceptions, attitudes and experiences of physical activity among second-generation South Asians in the UK, which potentially has importance for health and social inequalities policies in the UK.

## Abbreviations

UK: United Kingdom
CVD: Cardiovascular disease
NICE: National Institute for Health and Clinical Excellence
DASH study: Determinants of Adolescent Social well-being and Health study
MET: Metabolic Equivalent of Task
USA: United States of America

## References

[1] Fischbacher CM, Hunt S, Alexander L (2004). How physically active are South Asians in the United Kingdom? A literature review. J Public Health.

[2] Babakus WS, Thompson JL (2012). Physical activity among South Asian women: a systematic, mixed-methods review. Int J Behav Nutr Phys Act.

[3] Unit JHS (2006). Health Survey for England 2004: The Health of ethnic minority groups.

[4] Spring B, Moller AC, Colangelo LA, Siddique J, Roehrig M, Daviglus ML, Polak JF, Reis JP, Sidney S, Liu K (2014). Healthy Lifestyle Change and Subclinical Atherosclerosis in Young Adults: Coronary Artery Risk Development in Young Adults (CARDIA) Study. Circulation.

[5] Scarborough P, Bhatnagar P, Kaur A, Smolina K, Wickramasinghe K, Rayner M, Foundation BH (2010) Ethnic differences in cardiovascular disease.

[6] National Institute for Health and Clinical Excellence (2009) Methods for the development of NICE public health guidance (second edition). 27905711

[7] Duncan MJ, Al-Nakeeb Y, Nevill AM, Jones MV (2006). Body dissatisfaction, body fat and physical activity in British children. Int J Pediatr Obes.

[8] Duncan MJ, Woodfield L, Al-Nakeeb Y, Nevill AM (2008). Differences in physical activity levels between white and South Asian Children in the United Kingdom. Pediatr Exerc Sci.

[9] Duncan MJ, Birch S, Al-Nakeeb Y, Nevill AM (2012). Ambulatory physical activity levels of white and South Asian children in Central England. Acta Paediatr.

[10] Eyre EL, Duncan MJ, Smith EC, Matyka KA (2013). Objectively measured patterns of physical activity in primary school children in Coventry: the influence of ethnicity. Diabet Med.

[11] Falconer CL, Park MH, Croker H, Kessel AS, Saxena S, Viner RM, Kinra S (2014) Can the relationship between ethnicity and obesity-related behaviours among school-aged children be explained by deprivation? A cross-sectional study. BMJ Open. doi: http://dx.doi.org/10.1136/bmjopen-2013-003949 10.1136/bmjopen-2013-003949 PMC3902524 24413346

[12] Ghouri N, Purves D, McConnachie A, Wilson J, Gill JMR, Sattar N (2013). Lower cardiorespiratory fitness contributes to increased insulin resistance and fasting glycaemia in middle-aged South Asian compared with European men living in the UK. Diabetologia.

[13] Griffiths LJ, Cortina-Borja M, Sera F, et al. (2013) How active are our children? Findings from the Millennium cohort study. BMJ Open. doi: http://dx.doi.org/10.1136/bmjopen-2013-002893 10.1136/bmjopen-2013-002893 PMC3752053 23965931

[14] Harding S, Teyhan A, Maynard MJ, Cruickshank JK (2008). Ethnic differences in overweight and obesity in early adolescence in the MRC DASH study: The role of adolescent and parental lifestyle. Int J Epidemiol.

[15] Hayes L, White M, Unwin N, Bhopal R, Fischbacher C, Harland J, Alberti KGMM (2002). Patterns of physical activity and relationship with risk markers for cardiovascular disease and diabetes in Indian, Pakistani, Bangladeshi and European adults in a UK population. J Public Health Med.

[16] Hemmings S, Conner A, Maffulli N, Morrissey D (2011). Cardiovascular disease risk factors in adolescent British South Asians and whites: a pilot study. Postgrad Med.

[17] Karlsen S, Nazroo JY (2010). Religious and ethnic differences in health: evidence from the Health Surveys for England 1999 and 2004. Ethn Health.

[18] Khunti K, Stone MA, Bankart J, Sinfield PK, Talbot D, Farooqi A, Davies MJ (2007). Physical activity and sedentary behaviours of South Asian and white European children in inner city secondary schools in the UK. Fam Prac.

[19] Knight T, Smith Z, Lockton JA, Sahota P, Bedford A, Toop M, Kernohan E, Baker MR (1993). Ethnic differences in risk markers for heart disease in Bradford and implications for preventive strategies. J Epidemiol Community Health.

[20] Lean MEJ, Hans T, Anderson AS, Bradby H, Williams R, Han TS, Bush H (2001). Ethnic differences in anthropometric and lifestyle measures related to coronary heart disease risk between South Asian, Italian and general-population British women living in the west of Scotland. Int J Obes.

[21] McMinn AM, van Sluijs EMF, Nightingale CM, Griffin SJ, Cook DG, Owen CG, Rudnicka AR, Whincup PH (2011) Family and home correlates of children’s physical activity in a multi-ethnic population: The cross-sectional child heart and health study in england (CHASE). International Journal of Behavioral Nutrition and Physical Activity. doi: http://dx.doi.org/10.1186/1479-5868-8-11 10.1186/1479-5868-8-11 PMC3050786 21324105

[22] De Munter JS, Agyemang C, van Valkengoed IG, Bhopal R, Zaninotto P, Nazroo J, Kunst AE, Stronks K (2013). Cross national study of leisure-time physical activity in Dutch and English populations with ethnic group comparisons. Eur J Public Health.

[23] Owen CG, Nightingale CM, Rudnicka AR, Cook DG, Ekelund U, Whincup PH (2009). Ethnic and gender differences in physical activity levels among 9-10-year-old children of white European, South Asian and African-Caribbean origin: The Child Heart Health Study in England (CHASE Study). Int J Epidemiol.

[24] Pollard TM, Unwin N, Fischbacher C, Chamley JK (2008). Differences in body composition and cardiovascular and Type 2 diabetes risk factors between migrant and British-born British Pakistani women. Am J Hum Biol.

[25] Pollard TM, Hornby-Turner YC, Ghurbhurrun A, Ridgers ND (2012). Differences between 9–11 year old British Pakistani and White British girls in physical activity and behavior during school recess. BMC Public Health.

[26] Pomerleau J, McKeigue PM, Chaturvedi N (1999). Factors associated with obesity in South Asian, Afro-Caribbean and European women. Int J Obes.

[27] Riste L, Khan F, Cruickshank K (2001). High prevalence of type 2 diabetes in all ethnic groups, including Europeans, in a British inner city: relative poverty, history, inactivity, or 21st century Europe?. Diabetes Care.

[28] Smith NR, Kelly YJ, Nazroo JY (2012). The effects of acculturation on obesity rates in ethnic minorities in England: evidence from the Health Survey for England. Eur J Public Health.

[29] Williams R, Bhopal R, Hunt K (1994). Coronary risk in a British Punjabi population: Comparative profile of non-biochemical factors. Int J Epidemiol.

[30] Williams R, Shams M (1998). Generational continuity and change in British Asian health and health behaviour. J Epidemiol Community Health.

[31] Williams ED, Stamatakis E, Chandola T, Hamer M (2011). Physical activity behaviour and coronary heart disease mortality among South Asian people in the UK: an observational longitudinal study. Heart.

[32] Williams ED, Stamatakis E, Chandola T, Hamer M (2011). Assessment of physical activity levels in South Asians in the UK: Findings from the Health Survey for England. J Epidemiol Community Health.

[33] Yates T, Davies MJ, Gray LJ, Webb D, Henson J, Gill JM, Sattar N, Khunti K (2010). Levels of physical activity and relationship with markers of diabetes and cardiovascular disease risk in 5474 white European and South Asian adults screened for type 2 diabetes. Prev Med.

[34] Hine C, Fenton S, Hughes AO, Velleman G (1995). Coronary heart disease and physical activity in South Asian women: local context and challenges. Health Educ J.

[35] Williams ED, Nazroo JY, Kooner JS, Steptoe A (2010). Subgroup differences in psychosocial factors relating to coronary heart disease in the UK South Asian population. J Psychosom Res.

[36] Brophy S, Crowley A, Mistry R, Hill R, Choudhury S, Thomas NE, Rapport F (2011). Recommendations to improve physical activity among teenagers--a qualitative study with ethnic minority and European teenagers. BMC Public Health.

[37] Eastwood SV, Rait G, Bhattacharyya M, Nair DR, Walters K (2013). Cardiovascular risk assessment of south asian populations in religious and community settings: A qualitative study. Fam Pract.

[38] Farooqi A, Nagra D, Edgar T, Khunti K (2000). Attitudes to lifestyle risk factors for coronary heart disease amongst South Asians in Leicester: a focus group study. Fam Pract.

[39] Grace C, Begum R, Subhani S, Kopelman P, Greenhalgh T (2008) Prevention of type 2 diabetes in British Bangladeshis: qualitative study of community, religious, and professional perspectives. British Medical Journal. doi: 10.1136/bmj.a1931 10.1136/bmj.a1931 PMC2659954 18984633

[40] Horne M, Speed S, Skelton D, Todd C (2009). What do community-dwelling Caucasian and South Asian 60–70 year olds think about exercise for fall prevention?. Age & Ageing.

[41] Horne M, Skelton D, Speed S, Todd C (2010). The influence of primary health care professionals in encouraging exercise and physical activity uptake among White and South Asian older adults: Experiences of young older adults. Patient Educ Couns.

[42] Horne M, Skelton DA, Speed S, Todd C (2012). Attitudes and beliefs to the uptake and maintenance of physical activity among community-dwelling South Asians aged 60–70 years: A qualitative study. Public Health.

[43] Horne M, Skelton DA, Speed S, Todd C (2013). Perceived barriers to initiating and maintaining physical activity among South Asian and White British adults in their 60s living in the United Kingdom: a qualitative study. Ethn Health.

[44] Jepson R, Avan G, Bowes A, Harris F, Robertson R, Sheikh A (2008). Physical activity and black and minority ethnic groups: a qualitative study of South Asian people living in Scotland.

[45] Jepson R, Harris FM, Bowes A, Robertson R, Avan G, Sheikh A (2012). Physical Activity in South Asians: An In-Depth Qualitative Study to Explore Motivations and Facilitators. PLoS ONE.

[46] Johnson MRD (2000). Perceptions of Barriers to Healthy Physical Activity among Asian Communities. Sport, Education and Society.

[47] Khunti K, Stone MA, Bankart J, Sinfield P, Pancholi A, Walker S, Talbot D, Farooqi A, Davies MJ (2008). Primary prevention of type-2 diabetes and heart disease: Action research in secondary schools serving an ethnically diverse UK population. J Public Health.

[48] Pallan M, Parry J, Adab P (2012). Contextual influences on the development of obesity in children: A case study of UK South Asian communities. Prev Med.

[49] Rai DK, Finch H (1997). Physical activity from our point of view: qualitative research among South Asian and black communities.

[50] Rawlins E, Baker G, Maynard M, Harding S (2013). Perceptions of healthy eating and physical activity in an ethnically diverse sample of young children and their parents: the DEAL prevention of obesity study. J Hum Nutr Diet.

[51] Rogers A, Adamson JE, McCarthy M (1997). Variations in health behaviours among inner city 12-year-olds from four ethnic groups. Ethn Health.

[52] Victor CR (2014) Understanding Physical Activity in the Daily Lives of Bangladeshi and Pakistani Elders in Great Britain. ISRN Geriatrics 2014:8

[53] Sirard JR, Pate RR (2001). Physical Activity Assessment in Children and Adolescents. Sports Med.

[54] Nazroo JY (2003). The Structuring of Ethnic Inequalities in Health: Economic Position, Racial Discrimination, and Racism. Am J Public Health.

[55] Malterud K (2001). Qualitative research: standards, challenges, and guidelines. Lancet.

[56] Allender S, Cowburn G, Foster C (2006). Understanding participation in sport and physical activity amongst children and adults: a review of qualitative studies. Health Educ Res.

[57] Hosper K, Nierkens V, Nicolaou M, Stronks K (2007). Behavioural risk factors in two generations of non-Western migrants: do trends converge towards the host population?. Eur J Epidemiol.

[58] Hosper K, Nicolaou M, van Valkengoed I, Nierkens V, Stronks K (2011). Social and cultural factors underlying generational differences in overweight: a cross-sectional study among ethnic minorities in the Netherlands. BMC Public Health.

[59] Grieser M, Vu MB, Bedimo-Rung AL, Neumark-Sztainer D, Moody J, Young DR, Moe SG (2006). Physical activity attitudes, preferences, and practices in African American, Hispanic, and Caucasian girls. Health Educ Behav.

